# Body Esteem and Self-Efficacy of Pregnant Women with Gestational Diabetes Mellitus

**DOI:** 10.3390/ijerph20032171

**Published:** 2023-01-25

**Authors:** Agnieszka Bień, Agnieszka Pieczykolan, Magdalena Korżyńska-Piętas, Joanna Grzesik-Gąsior

**Affiliations:** 1 Chair of Obstetrics Development, Faculty of Health Sciences, Medical University of Lublin, 4/6 Staszica St., 20-081 Lublin, Poland; 2 State University of Applied Sciences in Krosno, 6 KazimierzaWielkiego St., 38-400 Krosno, Poland

**Keywords:** pregnancy, gestational diabetes mellitus, body esteem, generalized self-efficacy

## Abstract

The diagnosis of gestational diabetes mellitus provokes a change in a pregnant woman’s lifestyle, which may affect her well-being and precipitate a sense of loss of self-control over her own body. The perception of “body image” is not only physical appearance and physical attractiveness but also the emotional attitude to the body and beliefs about it. The aim of the study was to analyze the factors affecting body esteem and analyze the relationship between body esteem and self-efficacy in pregnant women with gestational diabetes mellitus. The study was conducted in the period from April 2019 to January 2021 among 287 women with gestational diabetes mellitus with the use of the following research tools: Body Esteem Scale (BES) and Generalized Self-Efficacy Scale (GSES). The explanatory variables for the sexual attractiveness variable were age (β = 0.252; *p* = 0.006) and education (β = 0.334; *p* = 0.007), for the weight concern variable were age (β = 0.161; *p* = 0.005), BMI (β = 0.334; *p* = 0.005), and education (β = 0.252; *p* = 0.033), for the physical condition variable, were age (β = 0.096; *p* = 0.004) and education (β = 0.213; *p* = 0.006). Positive correlations were found between self-efficacy and body esteem in the aspects of sexual attractiveness (*p* = 0.350), weight concern (*p* = 0.296), and physical condition (*p* = 0.286). Positive correlations were found between self-efficacy and body esteem in the aspects of sexual attractiveness (*p* = 0.350), weight concern (*p* = 0.296), and physical condition (*p* = 0.286). Older women who had better education and a lower BMI rated their bodies better. In women with gestational diabetes mellitus, high self-efficacy determines a better perception of their bodies in all areas: sexual attractiveness, weight concern, and physical condition.

## 1. Introduction

Gestational diabetes mellitus (GDM) is one of the common diseases accompanying pregnancy. It is described as a transient deterioration of glucose and insulin metabolism that exceeds the compensatory capacity of the pancreas, resulting in the development of clinically overt glucose intolerance. This disorder first appears in a given pregnancy or is first detected during pregnancy [1,2]. Achieving the glycemic values determined in healthy pregnant women is the goal of therapy. The basic element in the treatment of gestational diabetes mellitus is a diabetic diet and moderate, systematic physical activity. If it is difficult to achieve the recommended glucose levels, insulin therapy should be included [3]. GDM is one of the leading causes of complications for the mother and the developing fetus, such as pre-eclampsia, preterm labor, and fetal macrosomia. The prevalence of gestational diabetes mellitus is rapidly increasing worldwide. In Europe, the prevalence of GDM is 10.9%: the highest prevalence is observed in Eastern Europe at 31.5%, and the lowest in Northern Europe at 8.9%. In Poland, it is 6.2%. The prevalence in North America and the Caribbean is assessed as 7.1%, in South America and Central America 10.4%, and in Asian countries, it ranges from 1.2 to 49.5% [4].

Women who have had gestational diabetes mellitus in the past, in the future have several times higher risk of developing type 2 diabetes [5,6].

For every woman, pregnancy is a special moment in her life, during which she experiences many emotions, most often joy caused by the expectation of offspring. A woman’s functioning during pregnancy and her quality of life is affected by many aspects, such as her emotional state, health situation, socioeconomic status, involvement of her partner, or support from loved ones. The stage of pregnancy and its course—physiological or complicated—is also important [7]. The perception of pregnancy can be negatively affected by discomforts, such as nausea, vomiting, heartburn, bladder pressure, or back pain, which reduce the quality of life [7,8].

During pregnancy, a woman’s body undergoes dynamic changes in a relatively short period. The changes can put a strain on the mother-to-be not only physically but also psychologically. Significant weight gain may be perceived as a natural consequence of pregnancy, but in a situation of abnormal body perception and the conviction of a discrepancy between one’s appearance and the socially promoted ideal of a slim figure, a sense of loss of control and dissatisfaction with one’s body may arise. Moreover, this situation can create a risk of psychological distress for the pregnant woman, including low self-esteem, depressed mood and self-confidence, shame, anxiety, and even depression [7,8,9,10].

Women’s perceptions of pregnancy are also influenced by self-assessment and evaluation of their bodies. A pregnant woman has to adjust to her new situation and new position in society, as well as change the way she thinks about herself [7,8]. The perception of “body image” takes on different definitions, although researchers agree that it includes not only physical appearance and attractiveness but also other components such as emotional attitudes toward the body and beliefs about it [9].

Dissatisfaction with the appearance of one’s body reduces the sense of life satisfaction and self-confidence [11]. A woman’s body image during pregnancy can also be a predictor of postpartum body image and indicate a desire for rapid weight regulation during pregnancy, as well as after childbirth [12,13]. An abnormal perception of body image during pregnancy can negatively affect the formation of the mother–baby bond, as well as the desire to undertake breastfeeding [10].

The process of a woman’s adaptation to pregnancy, especially a complicated pregnancy, is related to, among other things, a sense of self-efficacy about her ability to handle the tasks and cope with the problems that arise during pregnancy. It is a person’s subjective sense of being able to bring about the desired situation through the actions taken [14,15].

### Purpose of the Study

The aim of the study was to analyze factors influencing body esteem and analyze the relationship between body esteem and self-efficacy in pregnant women with gestational diabetes mellitus.

## 2. Materials and Methods

### 2.1. Study Group

**Figure 1:**
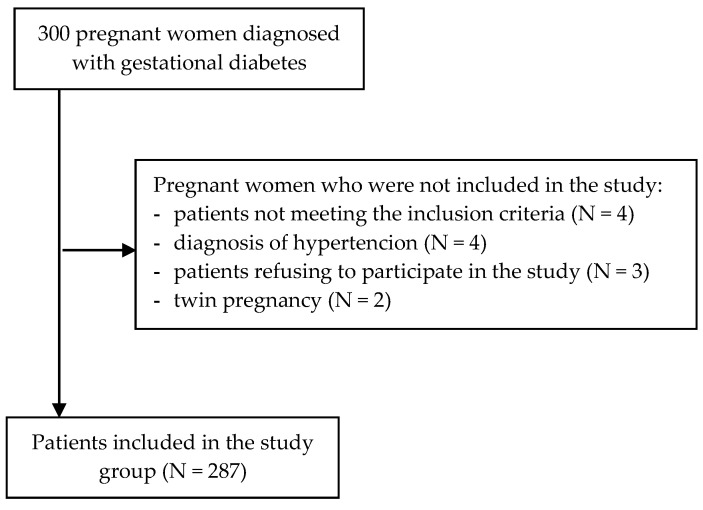
Flowchart of the recruitment process of the patients.

The study was conducted between April 2019 and September 2021 among patients of the pregnancy pathology department and gynecology–obstetrics outpatient clinics in Lublin (Poland). Two hundred and eighty-seven women with gestational diabetes mellitus took part in the study. Inclusion criteria for pregnant women in the study: the presence of gestational diabetes mellitus, consent to participate in the study, completed 18th year of life (the age of majority in Poland), Caucasian race, native language—Polish, single pregnancy, time from diagnosis of the disease at least 5 weeks, use of health care in Poland throughout the pregnancy. Exclusion criteria: age under 18, diagnosis of diabetes before pregnancy, multiple pregnancy, diagnosis of other diseases complicating pregnancy (e.g., hypertension, thyroid disease, threatened preterm labor), cognitive impairment, physical, mental, or sensory disability (Figure 1).

The Polish Society of Gynecologists and Obstetricians and Polish Diabetes Association criteria were used to diagnose GDM when any of the following criteria were met on OGTT: fasting blood glucose between 92–125 mg/dL, 60th-minute blood glucose ≥ 180 mg/dL, 120th-minute blood glucose between 153–199 mg/dL [16].

Based on the weight and height data of the respondents, their pre-pregnancy body mass index (BMI, Quetelet index) was calculated as weight in kilograms divided by height in meters squared. BMI classification was done according to the guidelines of the World Health Organization (WHO 2000) cut points: BMI < 18.5 kg/m^2^ (underweight); 18.5–24.99 kg/m^2^ (normal weight); 25.0–29.99 kg/m^2^ (overweight); and >30 kg/m^2^ (obese) [17].

Respondents were informed of the voluntariness and anonymity of their participation in the study, as well as the use of the results obtained for scientific purposes only. To conduct this study, the diagnostic survey method was used, a survey technique with the use of research tools:

The Body Esteem Scale (BES) developed by Franzoi and Shields (Polish adaptation by Lipowska and Lipowski) allows determining the respondents’ attitudes toward their bodies in three subscales: Sexual Attraction (SA), Weight Concern (WC), and Physical Condition (PC). The scale consists of 35 items that can be answered on a 5-point Likert scale, scoring from 1 to 5, where 1 means I have strong negative feelings, 5 means I have strong positive feelings, and 3 means a neutral attitude. The scale takes into account 3 areas related to self-assessment of one’s body: sexual attractiveness, weight concern, and physical condition. The score is obtained after adding all the points, and as the number of points increases, the body evaluation increases. The α-Cronbach coefficient of the Polish version of the test for women is 0.92, while the reliability coefficients for the individual factors were 0.76 for the sexual attractiveness subscale, 0.87 for the weight concern subscale, and 0.83 for the physical condition subscale [18].

The Generalized Self-Efficacy Scale, created by Schwarzer and Jerusalem, has been adapted to different segments of society for the concept of self-efficacy (Polish adaptation by Juczynski). It allows measuring the strength of an individual’s general belief in the effectiveness of coping with obstacles and difficult situations. It is designed to assess an individual’s beliefs about his or her ability to cope with difficult situations, so it can be used by people who are ill. GSES scale consists of 10 questions, in which there is a possibility of four answers scored from 1 to 4—the higher the score obtained by the respondent, the greater his sense of self-efficacy. The reliability of the test in the form of the α-Cronbach coefficient is 0.85 [19].

The standardized interview questionnaire included questions on the sociodemographic data of the respondents, including age, marital status, education, place of residence, family wealth, pregnancy planning, BMI, having children, and the current week of pregnancy.

The study was approved by the Lublin Medical University Bioethics Committee (approval no. KE-0254/166/2018). Respondents were informed that participation was voluntary, and that study results were anonymous and would be used exclusively for research purposes.

### 2.2. Statistical Analysis

The obtained results of the study were collected and statistically analyzed using STATISTICA 13.3. In the descriptive analysis, data were presented using mean value, standard deviation, frequency, and percentage. To examine differences in parameters appropriate statistical tests were used. Stepwise regression was used to identify predictors of BES scores. Stepwise regression is a method of regression model fitting in which the choice of predictive variables is performed using an automatic procedure. The correlation between quantitative variables was calculated. Pearson’s r-Pearson correlation was used to test the relationship between the selected variables, while the rho-Spearman correlation was used for ordinal variables. Regression analysis with moderating factors was performed to evaluate the variables. The study assumed a significance level of *p* < 0.05.

## 3. Results

Respondents’ Characteristics

Table 1 shows the characteristics of the women participating in the study. The average age of respondents was 28.84 ± 5.14 years. Among the 287 respondents participating in the study, 66.86% were married, with higher education (71.22%), living in the city (74.56%), assessing their family’s wealth as average (72.24%), planning a current pregnancy (67.59%), with a BMI indicating a normal weight (42.59%), and having no children (63.07%). The mean gestational age was 31.56 ± 3.61 weeks.

The results obtained from BES showed that among the three subscales: SA, WC, and PC, the female respondents rated their sexual attractiveness highest, while the lowest values were determined in the physical condition scale. In the study group, the average body score on the SA subscale was 46.43 ± 8.27, on the WC subscale it was 31.16 ± 8.45, and on the PC subscale it was 30.65 ± 6.65. The average level of GSES was 28.81 ± 5.63—Table 2.

Regression analysis showed that the explanatory variables for the variable SA were age (β = 0.252; *p* = 0.006) and education (β = 0.334; *p* = 0.007). It was shown that the body’s assessment of sexual attractiveness increased with age and was positively related to higher education. The adopted model explained 16.2% of the variance (F = 1.977; *p* < 0.05; R2 = 0.062).

The resulting regression model for the WC variable turned out to be a good fit to the data (F = 3.407; *p* < 0.05) and allowed explaining 14% of the variance of the dependent variable. The regression analysis showed that the statistically significant predictors in this model were age (β = 0.161; *p* = 0.005), BMI (β = 0.334; *p* = 0.005), and education (β = 0.252; *p* = 0.033). It was shown that body assessment of WC increased with age and body mass index and was positively associated with higher education.

Regression analysis showed that the explanatory variables for the PC variable were age (β = 0.096; *p* = 0.004) and education (β = 0.213; *p* = 0.006). It was shown that the body’s assessment of PC increased with age and was positively related to higher education. The adopted model explained 12.1% of the variance (F = 2.346; *p* < 0.05).

Regression analysis showed that the explanatory variables of GSES were age (β = 0.098; *p* = 0.014), wealth (β = −0.136; *p* = 0.024), and planning a pregnancy (β= 0.221; *p* = 0.046). It was shown that GSES self-efficacy increased with age and was positively associated with a better economic situation and the fact of planning the current pregnancy. The presented model was statistically significant, representing 9.4% of the variance (F = 2.249; *p* < 0.001)—Table 3.

## 4. Discussion

The diagnosis of gestational diabetes mellitus makes it necessary to change the pregnant woman’s lifestyle, and in some cases also to apply pharmacological treatment and hospitalization, which may affect her well-being, precipitate a sense of loss of self-control over her own body, and also affect the perception of the quality of life [20,21]. A review of the literature on pregnant women’s assessment of body image satisfaction shows conflicting results. On the one hand, some studies suggest higher body self-esteem during pregnancy, and acceptance of one’s body and physical changes associated with pregnancy. On the other hand, however, some women may experience anxiety and dissatisfaction with weight gain [22,23,24]. The aim of our study was to analyze factors influencing body esteem and analyze the relationship between body evaluation and self-efficacy in pregnant women with gestational diabetes mellitus.

Anatomical and physiological changes, as well as emotional changes which occur during pregnancy, affect women’s sex lives. As pregnancy progresses, the desire for intercourse and its frequency decreases [25,26,27]. In some women, fear of sexual activity may appear, which results from fear for the course of pregnancy, the health of the unborn child, and the inability to confront and expand knowledge on this subject with medical personnel [28]. Especially in the case of high-risk pregnancies, sexual activity is relegated to the background [29].

Women with complicated pregnancies place the functionality of their bodies above their physical appearance and attractiveness in terms of ensuring the health of the developing fetus. They, too, rate their appearance, as well as their sexual attractiveness, worse than women with uncomplicated pregnancies [26,30]. Interestingly, in our study, we showed that sexual attractiveness was the highest-rated component on the Body esteem scale. The reasons for this may include the fact that after the diagnosis of gestational diabetes mellitus, women began to follow a diet and take care of their physical activity, and these actions may have had a positive effect on their perception of their bodies, including their attractiveness.

Weight gain during pregnancy appears to be socially accepted, and thus far less “stigmatized” than at other times in a woman’s life [31,32]. In the situation of the diagnosis of gestational diabetes mellitus, and in view of the dynamics of “typical” changes occurring in the body of a pregnant woman, it is extremely important to monitor her weight. Both excessive and insufficient weight gain require special attention in this group of women. Nutritional therapy and exercise interventions to control blood glucose levels should be used to control the recommended weight gain during pregnancy to reduce possible adverse pregnancy outcomes [33]. Hodgkinson et al. showed that many women have unrealistic expectations about their body appearance during the perinatal and postpartum periods, leading to their dissatisfaction with their bodies and feeling worse about themselves [34]. Mothers with higher levels of dissatisfaction with their bodies show lower well-being, lower self-esteem, and perceived parenting competence [26].

A growing number of studies have shown a negative relationship between pre-pregnancy BMI and weight gain in relation to body satisfaction during pregnancy [35,36]. The results of our study also showed such a relationship. The level of body image evaluation in the weight concern domain of pregnant women with gestational diabetes mellitus was found to be related to their BMI level—with a decrease in BMI, body image satisfaction and self-esteem increased. Erkaya et al. showed a negative relationship between pregnant women’s body image and age, pre-pregnancy weight, physical self-perception, and satisfaction with their current weight [37]. In contrast, Shloim et al. noted that despite normal body weight, some pregnant women presented abnormal perceptions of their bodies, which was due to their attitudes toward their pre-pregnancy bodies [38]. A meta-analysis by Hodgkinson et al. showed that pregnant women perceived changes in their bodies as typical and inevitable for pregnancy, but did not accept “being obese”, viewing their bodies as foreign. This situation generated the need to control the increase in body weight and the emergence of a sense of anxiety when the weight gain was too large or too small [34].

Undertaking physical activity by pregnant women has many health benefits for the mother and for the course of pregnancy, reducing the risk of abnormal pregnancy course [39]. The physical activity of pregnant women should be primarily aimed at improving the fitness of the body. Among other things, it prevents excessive fetal and maternal weight gain, makes gestational diabetes mellitus more manageable, and reduces the number of cesarean sections [40]. Despite these positive effects, studies have shown that women tend to reduce their level of physical activity during pregnancy, mainly as a result of fear of miscarriage, but also due to a lack of motivation and desire to take up exercise [41].

A meta-analysis by Mijatovic-Vukas et al. found that physical activity performed before pregnancy reduces the risk of gestational diabetes mellitus by 22–86%, while the degree of potential protection depends on the duration and type of physical activity [42]. The combination of diet and exercise reduces excessive weight gain, which is directly linked to the development of GDM, as well as improving insulin sensitivity [43,44]. Pullmer et al. found that health-related habit strength in women of reproductive age may offer protection against low levels of body satisfaction during pregnancy [24].

Our study shows that older women with better education rated their bodies better in each component of the Body Esteem Scale. These data are supported by the literature. An analysis by Cevik et al. showed a higher level of self-esteem in a group of post-graduate and working women. In addition, women whose husbands showed a negative attitude toward weight gain during pregnancy showed a negative correlation between the level of self-assessment of their body image and depression, and a positive correlation between the level of self-assessment and their body image [45]. The relationship between the level of education and body evaluation can be explained by the influence of better education on the need for lifestyle adjustments and modifications after the diagnosis of gestational diabetes mellitus, as well as the greater health awareness of these women.

Self-efficacy is a cognitive process by which a person evaluates his or her ability to cope with various situations. It is an important predictor of attitudes, emotions, and behavior of pregnant women [15,46]. The diagnosis of gestational diabetes mellitus in women compounds social and psychological problems and causes anxiety about themselves as well as their baby. On the one hand, the diagnosis results in an increased sense of control over eating habits and weight gain, which translates into taking responsibility for one’s own and the baby’s health. However, an important aspect is the lack of knowledge and understanding of the causes of GDM and the conflicting information received from medical personnel, which greatly limits the process of self-control and self-efficacy [47].

In our study, the average level of self-efficacy in the group of pregnant women was 28.47, which was within the upper limits of the average reference values. Pregnant women diagnosed with gestational diabetes mellitus having a higher level of self-efficacy will be more likely to treat the situation as a challenge, more likely to seek an effective way of coping with the difficulties encountered, and the necessity of the therapeutic process will motivate them to act and engage in the behaviors undertaken, requiring them to change their lifestyle, among other things. These pregnant women will also feel greater satisfaction with the tasks undertaken and carried out [20,48].

According to our study, such behavior will be exhibited by older women and those whose pregnancy was planned. These pregnant women are convinced of their competence and are aware that their effort will be necessary to effectively perform the task. This is particularly important because, as studies show, a diagnosis of GDM can increase a woman’s anxiety, lead to a negative perception of her health, and the occurrence of less positive pregnancy experiences compared to women with a normal course of pregnancy [49].

Having financial resources is important during diabetes treatment. Better livelihoods allow for a greater ability to cope with difficult situations and challenges which may occur during the disease [50]. Diagnosed diabetes requires expenditures on tests, food, and medicines, which can translate into financial resources for the family, and the same can negatively affect the mental state of women [51]. Our research has shown that poorer self-esteem about one’s wealth is related to lower self-efficacy. Decreasing self-efficacy is a risk factor for decreased motivation to undertake adaptive activities to the problems at hand, passivity, helplessness, and even the onset of depression and/or anxiety [19,48]. The literature shows that pregnant women with low self-efficacy are more likely to prefer delivery by cesarean section [52]. In contrast, Ma et al. found that women with a high sense of self-efficacy were more able to cope with symptoms of prenatal anxiety, adapt more easily to physical discomforts that arise, as well as better prepare for childbirth [53]. A study by Schwartz et al. found higher levels of self-efficacy in women with subsequent pregnancies, due to their experience of pregnancy and childbirth and their perception of themselves as more competent [52].

In our study, a positive relationship between body evaluation in each of the analyzed components and the self-efficacy of respondents with gestational diabetes mellitus was observed. On the other hand, Kumcagız et al. showed that there was a positive correlation between self-esteem and body image [54]. In the context of these results, it can be concluded that a higher sense of self-efficacy is a significant predictor affecting higher body evaluation of pregnant women. It acts, as it were, to protect against the occurrence of negative consequences of low body evaluation, such as emotional disorders, anti-health behaviors, excessive weight gain during pregnancy, and eating disorders [10,24,55].

Assessment of body image during pregnancy should be an important part of prenatal care, as it allows for the skillful education of women on how to cope with the physiological changes that occur during pregnancy. This is especially true for women who are dissatisfied with their bodies, in whom pregnancy exacerbates negative appraisal and leads to the introduction of dietary restrictions, increased exercise, or psychological problems, such as depression [24,56]. Thus, an important part of the care of pregnant women with gestational diabetes mellitus is to conduct education, during which knowledge will be imparted about the use of an appropriate diet, the need to maintain optimal body weight, and to maintain or undertake physical activity [57]. Actions taken towards pregnant women to improve their body image would enable them to have a more positive perception of pregnancy, and a better quality of life, and this would have a positive impact on their mental health, especially when pregnancy is complicated by gestational diabetes mellitus.

The possibility of gaining greater satisfaction with one’s body during pregnancy is an important research perspective that may shed new light on the processes underlying potential maternity-induced identity change. Further research aimed at pregnant women’s body esteem is needed to further our understanding of the nature, course, and possible adaptive mechanisms of body image during pregnancy.

### Strengths and Limitations

The study provides information on the relationship between body esteem and self-efficacy among women with pregnancies complicated by gestational diabetes mellitus, which adds value to the rather poor literature on the analyzed topic. The results of the study can be used for more effectively planned informational and emotional support for pregnant women with gestational diabetes mellitus based on a better understanding of their specific and heterogeneous needs. The use of standardized research tools, which will enable other researchers to continue the study, to compare results and draw conclusions is an important strength of the study.

Our study is not free of limitations. The study was conducted in one province in Poland, so our sample may not be representative of the general population of pregnant women. It was a cross-sectional study, which limits our understanding of the predictors affecting the body evaluation of pregnant women with gestational diabetes mellitus. Our study would have been complemented by a longitudinal study to uncover causal relationships between the variables studied. In our study, we did not examine pregnant women’s health behaviors, including women’s nutrition and physical activity, which would have allowed a deeper analysis of the issue.

## 5. Conclusions

Predictors associated with body image in pregnant women with gestational diabetes mellitus are age and education in the areas of sexual attractiveness and physical condition and age, BMI, and education in the weight concern component. Older women, who have better education, and have a lower BMI assess their bodies better.

In women with gestational diabetes mellitus, self-efficacy is crucial to shaping body image—a high sense of self-efficacy determines a better perception of one’s body in all areas: sexual attractiveness, weight concern, and physical condition.

## Figures and Tables

**Table 1:** Characteristics of the study group—pregnant women with diabetes.

Parameters		n	%
Mean age (SD)	28.84 (±5.14), range 19–41	---	---
Marital status	single	14	4.94
	married	192	66.86
	partnership	81	28.20
Education	primary/professional training	6	2.03
	secondary	77	26.74
	university degree	204	71.22
Place of residence	city	214	74.56
	country	73	25.44
Family wealth	very rich	16	5.52
	rather rich	51	17.73
	average	207	72.24
	rather poor	13	4.51
Planned pregnancy	yes	194	67.59
	no	93	32.41
BMI	normal	122	42.59
	overweight	108	37.50
	obesity	57	19.91
Having children	no	181	63.07
	yes	106	36.93
Mean gestational age in weeks (SD)	31.56 (±3.61), range 25–39	---	---

**Table 2:** Body esteem and self-efficacy of pregnant women.

Categories		M	SD	Me	Min	Max
BES	SA	46.43	8.27	46.00	29.00	65.00
	WC	31.16	8.45	30.00	13.00	50.00
	PC	30.65	6.65	30.00	19.00	45.00
GSES		28.81	5.63	30.00	10.00	40.00

BES—Body Esteem Scale; SA—Sexual Attraction; WC—Weight Concern; PC—Physical Condition. GSES—Generalized Self-Efficacy Scale; M—mean; SD—standard deviation; Me—median.

**Table 3:** Regression analysis for body evaluation and self-efficacy in women with gestational diabetes mellitus.

**Predictors**	**SA** **F = 1.977; *p* < 0.05; R^2 =^ 0.162**				
	**B**	**SE**	**β**	***t***	***p***
(Constant)	38.617	5.924		6.519	0.000
Age	0.084	0.182	0.252	0.459	0.006
Education	5.818	2.117	0.334	2.748	0.007
**Predictors**	**WC** **F = 3.407; *p* < 0.05; R^2 =^ 0.140**				
	**B**	**SE**	**β**	***t***	***p***
(Constant)	39.918	5.470		7.279	0.000
Age	0.098	0.185	0.161	0.528	0.005
BMI	0.116	1.995	0.334	2.882	0.005
Education	4.495	2.071	0.252	2.170	0.033
**Predictors**	**PC** **F = 2.346; *p* < 0.05; R^2^= 0.121**				
	**B**	**SE**	**β**	***t***	***p***
(Constant)	25.068	4.350		5.763	0.000
Age	0.125	0.149	0.096	0.832	0.004
Education	2.991	1.620	0.213	1.846	0.006
**Predictors**	**GSES** **F = 2.249; *p* < 0.001; R^2^= 0.094**				
	**B**	**SE**	**β**	***t***	***p***
(Constant)	25.244	3.909		6.458	0.000
Age	0.107	0.129	0.098	0.824	0.014
Perceived family wealth	−1.628	1.378	−0.136	−1.182	0.024
Planned pregnancy	2.548	1.357	0.221	1.878	0.046

SA—Sexual Attraction; WC—Weight Concern; PC—Physical Condition; GSES—Generalized Self-Efficacy Scale BMI—Body Mass Index; Test–Revised: B—unstandardized regression coefficient; SE—bootstrapped standard errors; β—standardized coefficients; Student’s t-Tests; *p*-value.

## Data Availability

The data presented in this study are available on request from the corresponding author.
